# Blocking Notch-Signaling Increases Neurogenesis in the Striatum after Stroke

**DOI:** 10.3390/cells9071732

**Published:** 2020-07-20

**Authors:** Giuseppe Santopolo, Jens P. Magnusson, Olle Lindvall, Zaal Kokaia, Jonas Frisén

**Affiliations:** 1 Department of Cell and Molecular Biology, Karolinska Institute, SE-171 77 Stockholm, Sweden; giuseppe.santopolo@ki.se (G.S.); jens.magnusson@ki.se (J.P.M.); 2 Lund Stem Cell Center, University Hospital, SE-221 84 Lund, Sweden; olle.lindvall@med.lu.se (O.L.); zaal.kokaia@med.lu.se (Z.K.)

**Keywords:** neurogenesis, astrocyte, stem cell, stroke, striatum

## Abstract

Stroke triggers neurogenesis in the striatum in mice, with new neurons deriving in part from the nearby subventricular zone and in part from parenchymal astrocytes. The initiation of neurogenesis by astrocytes within the striatum is triggered by reduced Notch-signaling, and blocking this signaling pathway by deletion of the gene encoding the obligate Notch coactivator Rbpj is sufficient to activate neurogenesis by striatal astrocytes in the absence of an injury. Here we report that blocking Notch-signaling in stroke increases the neurogenic response to stroke 3.5-fold in mice. Deletion of Rbpj results in the recruitment of a larger number of parenchymal astrocytes to neurogenesis and over larger areas of the striatum. These data suggest inhibition of Notch-signaling as a potential translational strategy to promote neuronal regeneration after stroke.

## 1. Introduction

Every year, 13.7 million people worldwide suffer a stroke, of whom 5.5 million die and as many suffer permanent loss of function with debilitating outcomes [1]. Effective treatments to promote cell regeneration in the chronic post-ischemic phase are lacking. In most parts of the brain, such as the cortex, there is no apparent neuronal replacement after stroke [2]. In the striatum, however, there is a regenerative response resulting in the replacement of a small proportion of the lost neurons [3,4].

Neurogenesis is abundant in the subventricular zone and in the dentate gyrus of the hippocampus in rodents and many other mammals. In the subventricular zone, neural stem cells differentiate into transit amplifying progenitor cells, which then mature into neuroblasts, identified through the markers Doublecortin (Dcx) and Polysyalilated Neuronal Cell Adhesion Molecule (PSA-NCAM). The neuroblasts migrate to the olfactory bulbs, where they differentiate into mature neurons. In the dentate gyrus, neural stem cells are largely quiescent, and 1-2% of them proliferate [5,6,7]. These divisions generate transit amplifying cells, which proliferate to actively generate neuroblasts. Neurogenesis has been identified in other brain regions as well, but, in these non-canonical areas, it is either a sporadic event, or it is not conserved between different species. Striatal neurogenesis has been observed under physiological conditions in the striatum of rabbits, rats, and non-human primates [3,8,9,10]. Humans are no exception, and neurogenesis has been found in the subventricular zone [11,12], dentate gyrus [13,14], and striatum [15].

After an ischemic event, neuroblasts double-positive for the markers Dcx and PSA-NCAM, or mature neuronal markers like NeuN, emerge in the striatum [3,4]. It is estimated that around 80-90% of neuroblasts generated following a stroke will not mature into neurons, but these neuroblasts seem to have a role in the recovery process as their ablation leads to impaired recovery [16].

The presence of a regenerative program in the striatum poses the question of whether it is possible to enhance this process to promote recovery. The new neurons in the striatum derive, in roughly equal proportions, from the nearby neurogenic subventricular zone and from parenchymal astrocytes [17]. Reduced Notch-signaling in parenchymal astrocytes after stroke triggers them to enter a neurogenic program, and blocking Notch-signaling by deleting the key downstream transcription factor Rbpj results in neurogenesis by striatal astrocytes in the absence of any insult [17]. Blocking Notch-signaling in striatal astrocytes initiates a transition to a neural stem cell-like state, from which some of the cells progress to transit amplifying divisions, giving rise to clusters of progenitor cells, which, in turn, generate neuroblasts [18]. Although reduced Notch-signaling is necessary and sufficient to trigger neurogenesis by parenchymal astrocytes, it is only a minority of the astrocytes in the stroke area that gives rise to new neurons. Based on transcriptome analysis, there are no apparent subclasses of astrocytes within the striatum that could explain why only some enter the neurogenic lineage [18], raising the question of whether additional suppression of Notch-signaling would recruit more astrocytes to neurogenesis. Astrocytes represent a significant proportion of all cells in the brain and are dispersed throughout the tissue, making them an attractive source for in vivo generation of new neurons.

In the present study, we assessed whether a further reduction of Notch-signaling in astrocytes could promote a stronger and more widespread neurogenic response to a striatal stroke. We report that deleting *Rbpj* specifically in astrocytes results in an approximate doubling of the number of cells that enter the neurogenic program after a striatal stroke, with a concomitant increase in neuroblasts. The neurogenic response is also more widespread in the stroke-afflicted striatum when Notch-signaling is blocked. This demonstrates that the neurogenic response after stroke can be substantially promoted by recruiting more parenchymal astrocytes to neurogenesis, identifying a new potential therapeutic target.

## 2. Materials and Methods

### 2.1. Animals

All experimental procedures were carried out in compliance with Swedish laws and the ethical rules by the home institution (Karolinska Institutet, Stockholm, Sweden). The ethical permit was reviewed and approved by Stockholms Norra Djurförsöksetiska Nämnd and the Malmö-Lund Ethical Committee. Mice were maintained on a 12 h light/dark cycle with access to food and water ad libitum. C57Bl6 Cx30-CreER; Rbpj^fl/fl^; R26-tdTomato mice, at least two months old, were used for the experiments.

### 2.2. Tamoxifen Injection

Animals were administered 2 mg tamoxifen/day via intraperitoneal injection for five consecutive days. Tamoxifen was dissolved in 99% ethanol (1:10) in corn oil. Days were counted from the last day of injection.

### 2.3. Middle Cerebral Artery Occlusion

All experimental procedures were approved by the Malmö-Lund Ethical Committee. Middle cerebral artery occlusion was performed using the protocol by Andsbeg, Kokaia, and Lindvall, 2001 [19]. Briefly, anesthesia was induced by placing the mice in an induction box and exposing them to an air solution of 4% isoflurane (Baxter, FDG9623). All animals were locally injected with Marcaine for pain relief (20 μL of 2.5 mg/mL stock solution, Astra Zeneca). Anesthesia efficiency was assessed by loss of the paw reflex. The mice were moved to the surgery table and kept under anesthesia by inhalation of 1.5-2% isoflurane. Throughout the procedure, brain blood flow was monitored using a laser Doppler. The animals were moved to a supine position and an incision was performed on the neck. The common and external carotid arteries were ligated, respectively, and the internal carotid artery was clipped temporarily with a metal microvessel clip. An incision was made in the internal carotid artery and a silicon-coated filament was inserted and pushed all the way to the clip, which was at this point removed to allow the filament to be further pushed to the middle cerebral artery. The filament was left in the middle cerebral artery for 35 min and then removed to allow reperfusion. After the removal of the filament, the internal carotid artery was permanently ligated. The necks of the mice were sutured and the animals were allowed, under supervision, to wake from anesthesia and placed back in the cage.

### 2.4. Immunofluorescence

Immunofluorescence was performed as previously described [17]. Briefly, brains were sectioned at a thickness of 30 µm and left 1 h in blocking solution (10% normal donkey serum, 0.3% Triton-X1000, 0.04% NaN_3_ in PBS). Following the blocking step, the sections were incubated with primary antibodies (diluted in blocking solution) overnight at 4 °C. The primary antibodies used were rabbit anti-Ascl1 (Cosmo Bio Co., CAC-SK-T01-003, concentration 1:250), goat anti-Dcx (Santa Cruz, SC-8066, concentration 1:500), mouse anti-NeuN (Millipore, MAB377, concentration 1:500), rabbit anti-S100β (DAKO, Z0311, concentration 1:200, antibody was directly conjugated with fluorophore Alexa-647) and rat anti-Ki67 (eBioscience, 14-5698, concentration 1:1000). Subsequently, the sections were washed three times for 5 min in PBS before incubation with secondary antibodies (1:500 in blocking solution) against the species for the specific primary antibodies and conjugated with the fluorophores Alexa-488, Cy3 and Alexa-647. For Ascl1 detection we used a Tyramide Signal Amplification (TSA) protocol (PerkinElmer, NEL701001KT).

## 3. Results

### 3.1. Blocking Notch-Signaling Results in a Larger Number of Neuroblasts after Stroke

Astrocytes in the striatal parenchyma generate new neurons after stroke, as well as following ablation of *Rbpj* [17]. We asked whether combining *Rbpj* deletion and stroke could increase the generation of neuroblasts, or if the two conditions individually bring the neurogenic potential of striatal astrocytes to its full capacity.

**Figure 1:**
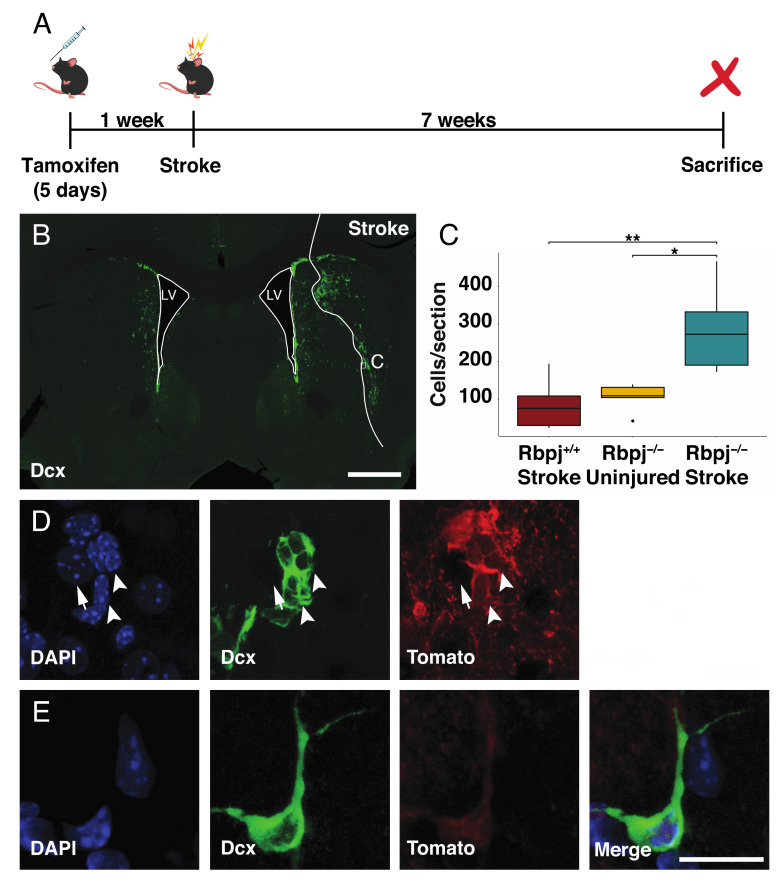
An increased number of neuroblasts is generated after stroke by blocking Notch-signaling. Analysis of Dcx expressing cells in the brain of mice with selective ablation of *Rbpj* in astrocytes after stroke: (**A**) Summary of the experimental procedure—mice were administered tamoxifen via intraperitoneal injection for five consecutive days. One week after the last injection, stroke was induced, and the animals were then sacrificed seven weeks later; (**B**) Overview of Dcx^+^ cells in the injured and uninjured hemisphere seven weeks after stroke (scalebar = 500 µm); (**C**) Quantification of the number of Dcx^+^/Tomato^+^ cells. For each animal, four sections around the core of the lesion have been selected and quantified. The average number of cells has been compared between the injured and the uninjured hemisphere from the same animals (*p* = 0.041) and with the injured hemisphere of wild-type mice (*p* = 0.005). Data were analyzed using a paired t-test, and results are presented as mean ± SD; (**D**,**E**) Close-up of clusters; (**D**) Single close-up; (**E**) Dcx^+^/Tomato^+^ cells found in the penumbra of the stroke area. Arrowheads indicate cells that are positive for both Dcx and Tomato, while the arrow points at a Tomato+ cell (scalebar = 15 µm and 6 µm respectively in **D** and **E**).

We induced conditional disruption of the gene for the obligate Notch coactivator Rbpj by administering tamoxifen to Cx30-CreER; Rbpj^fl/fl^; R26-tdTomato mice, and a week later we induced an ischemic stroke using the middle cerebral artery occlusion model. Cx30-CreER allowed recombination specifically in astrocytes, and the reporter allele enabled identification of the recombined cells and their progeny [20]. The mice were sacrificed seven weeks after the stroke, and the uninjured side, contralateral to the stroke, from the same animals served as the control (Figure 1A).

Neuroblasts are newly generated immature neurons, which can be identified by the expression of the marker Dcx. Tissue sections were stained for Dcx (Figure 1B), and Dcx^+^/Tomato^+^ cells were counted (Figure 1C). The number of Tomato^+^ neuroblasts was significantly higher in the striatum on the stroke side after deletion of *Rbpj* in astrocytes (270 ± 120 cells/section; mean+SD; *n* = 5) compared with the contralateral uninjured side of the same animals (110 ± 40 cells/section; *n* = 5; *p* = 0.041) or with the stroke side from *Rbpj*^+/+^ animals (76 ± 64 cells/section; *n* = 7 *p* = 0.005). These findings demonstrated that the combination of stroke and blocking Notch-signaling increased the number of neuroblasts 2.5- and 3.5-fold, respectively, compared to either blocking Notch-signaling or stroke alone. Blocking Notch-signaling is thus an efficient way to promote neurogenesis after stroke.

Clusters of neuroblasts appeared in more lateral positions within the striatum compared to the uninjured side in mice in which Notch-signaling had been blocked in astrocytes (Figure 1D). There were also individual neuroblasts, which lacked a clear migratory morphology (Figure 1E).

Neuroblasts from parenchymal astrocytes give rise to mature striatal interneurons after stroke or *Rbpj* deletion [17]. At the seven-week time point after the stroke that we analyzed in this study, there were only a few astrocyte-derived cells that became mature neurons. The relative number of NeuN+ neurons between the different conditions largely mirrored that of neuroblasts (Figure 1C), although there were no statistically significant differences in the number of mature neurons at this early time point: *Rbpj^-/-^* and stroke (12 ± 7 cells/section; mean ± SD), the uninjured hemisphere of the same animals (5 ± 2 cells/section; mean ± SD; *p* = 0.125), and *Rbpj^+/+^* and stroke (6 ± 9 cells/section; mean ± SD; *p* = 0.145; Figure S1).

### 3.2. Recruitment of Parenchymal Astrocytes to the Neurogenic Lineage by Notch-Signaling Blockade in Stroke

**Figure 2:**
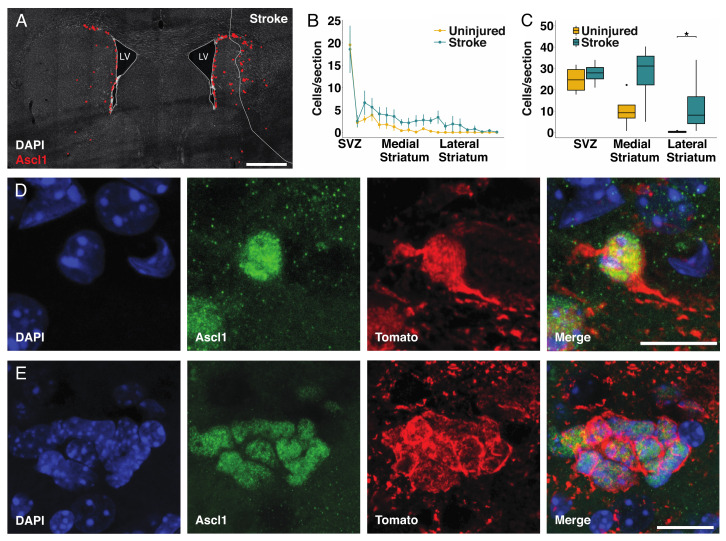
Astrocytes are more efficiently recruited to the neurogenic lineage when blocking Notch-signaling: (**A**) Overview of transient amplifying progenitor cells in the uninjured and injured hemisphere seven weeks after stroke. Red dots are positioned to overlap with Ascl1^+^ cells for better visualization (scalebar = 500 µm); (**B**) Analysis of the number of Ascl1^+^/Tomato^+^ cells in animals with deletion of *Rbpj* specifically in astrocytes. For each animal, four sections spanning throughout the lesion have been selected, and the average number of cells per section has been compared with the uninjured hemisphere from the same animal. To make the data comparable between animals, the distance from the striatum has been converted in percentage coordinates, where 0 represents the subventricular zone (SVZ), and 100 represents the lateral end of the striatum. The transition between the SVZ and medial striatum was placed at 10% and the one between medial and lateral striatum at 50%. The number of Ascl1^+^ cells in the stroke side is significantly higher (paired t-test, mean ± SEM; *p* = 0.028); (**C**) The number of transient amplifying progenitor cells in the lateral striatum is significantly higher in the stroke side compared to the uninjured (the data were analyzed using a paired two-way ANOVA, which allows the concomitant analysis of number and location of cells of interest within the striatum; data are presented as mean ± SD *p* = 0.041); (**D**,**E**) Examples of single cells; (**D**) Examples of clusters; (**E**) Ascl1^+^/Tomato^+^ cells found in the penumbra of the stroke area in different animals (scalebar = 10 µm in **D** and 15 µm in **E**).

An early marker for the recruitment of parenchymal astrocytes to the neurogenic lineage after stroke or after deletion of *Rbpj* is the expression of the proneural transcription factor Ascl1 in astrocytes [17]. We previously described the transformation process from astrocyte to Ascl1 expressing cell [17], and representative pictures are provided in Figure S2 and Figure S3. In brief, single Ascl1^+^ cells initially maintained a typical astrocyte morphology (Figure 2D, Figure S2A,B and Figure S3A–E). During the first stages of differentiation into transit amplifying progenitor cells, Ascl1^+^ astrocytes maintained the expression of the astrocyte marker S100β (Figure S2A,B). Subsequently, these cells lost their processes and assumed a round morphology reminiscent of transit amplifying progenitor cells, while maintaining the expression of S100β during the first divisions (Figure S2C,D). During this phase, the neurogenic astrocytes expressed the cell proliferation marker Ki67 (Figure S3A–E) and formed clusters of proliferating cells (Figure S3F–H). We found that the number of Ascl1+ cells was higher in the striatum, not including the subventricular zone, of the stroke side (121 ± 57cells/section; *n* = 5) compared to the contralateral uninjured striatum with *Rbpj* deletion only (37 ± 25/section; *n* = 5, *p* = 0.015) (Figure 2A).

There was no significant difference in the number of Ascl1+/Tomato+ cells in the subventricular zone (*p* = 0.486) or in the medial striatum (*p* = 0.2), whereas the combination of stroke and Notch-signaling blockade led to a >40-fold increase in the number of Ascl1-expressing astrocytes in the lateral half of the striatum, with many of these cells residing in the stroke penumbra (Figure 2B,C). This was noteworthy as stroke or deletion of *Rbpj* alone resulted in the recruitment of astrocytes to the neurogenic lineage almost exclusively in the medial striatum [17].

Ascl1^+^/Tomato^+^ cells were observed as singlets or in clusters, even in the lateral striatum of the injured hemisphere (Figure 2D,E). Single Ascl+ cells with astrocyte morphology (Figure 2D) as well as clusters of tightly packed Ascl1+ cells (Figure 2E) are indicative of activation of local astrocytes, followed by transit amplifying divisions, rather than migration of cells from, for example, the subventricular zone into the area [17].

### 3.3. Blocking Notch-Signaling Increases the Number of Transient Amplifying Progenitor Cell Clusters

An important question is whether the deletion of *Rbpj* leads to an increase in the number of astrocytes gaining neurogenic potential following stroke or if it induces the same subpopulation of cells to proliferate further. We previously reported how blocking Notch-signaling in astrocytes induces a neurogenic program, which follows the sequential steps of induction of Ascl1 expression, followed by several cycles of proliferation and, subsequently, commitment to the neuronal lineage with neuroblast differentiation [17]. We observed how neurogenic astrocytes in the mouse striatum generated clusters of transient amplifying progenitor cells and neuroblasts. Each cluster derived from a single neurogenic cell that proliferated several times. Thus, by quantifying the number of clusters, we could infer the number of astrocytes that became neurogenic [17].

**Figure 3:**
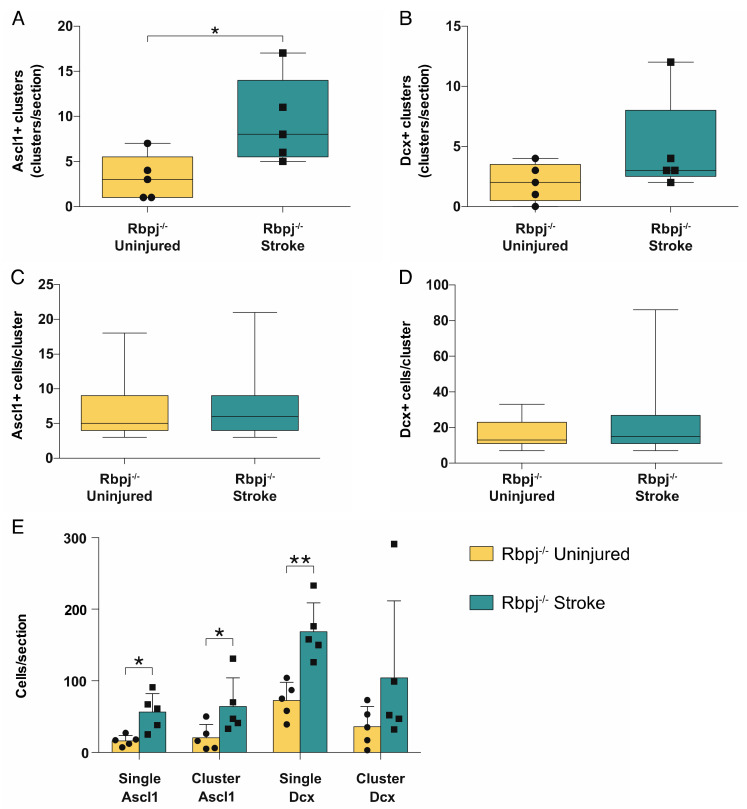
Analysis of cluster number and size. Analysis of the Ascl^+^ and Dcx^+^ clusters in the stroke side compared to the uninjured striatum: (**A**,**B**) Quantification of the total number of clusters. Groups of Ascl1^+^ cells have been defined as clusters only when found in contact with each other and in numbers ≥3 cells; Dcx^+^ cells had to be in groups ≥7 to be considered a cluster. The number of Ascl1^+^ clusters was significantly higher in the stroke side compared to the uninjured striatum (*p* = 0.014); (**C**,**D**) Analysis of the size of Ascl1^+^ and Dcx^+^ cells in the stroke and uninjured striatum; (**E**) Analysis of the total number of cells found in clusters and as singlets. Ascl1^+^/Tomato^+^ cell numbers are increased both as single cells (*p* = 0.022) and in clusters (*p* = 0.023), while there is a significant increase only in the number of Dcx^+^/Tomato^+^ neuroblasts singlets (*p* = 0.0097). The data were analyzed using a paired t-test and are presented as mean ± SD.

We quantified the number and size of Ascl1^+^/Tomato^+^ and Dcx^+^/Tomato^+^ clusters. The rationale was that an increase in the number of neurogenic astrocytes would lead to the observation of more clusters, while an increase in the number of cells per cluster would indicate that each neurogenic astrocyte underwent more proliferative cycles. There was a significant increase in the number of Ascl1+ cell clusters on the stroke side (9.4 ± 4.8 clusters/section; mean+SD) compared to the uninjured side (3.2 ± 2.5 clusters/section; *p* = 0.014) (Figure 3A). The difference in the number of neuroblast clusters corresponded in magnitude to that of the Ascl1+/Tomato+ clusters, but there was higher variability and no significant difference (4.8 ± 4.1 vs. 2.0 ± 1.6 clusters/section; *p* = 0.18) (Figure 3B).

We next quantified the number of cells composing each cluster. This number did not differ either for Ascl1+/Tomato+ transient amplifying progenitor cells (6.6 ± 3.6 vs. 7.0 ± 4.0 cells/cluster; mean+SD *p* = 0.34) or for neuroblasts (17 ± 73 vs. 22 ± 17 cells/cluster; *p* = 0.36) (Figure 3C,D).

Finally, we compared the number of transient amplifying progenitor cells and neuroblasts that could be seen as singlets or in clusters in the injured hemisphere compared to the uninjured side. The numbers of transient amplifying Ascl1+/Tomato+ progenitor cells, both as single cells and clustered, were significantly increased in the stroke side (56 ± 26 vs. 16 ± 7 single cells/section; *p* = 0.022; 64 ± 40 vs. 21 ± 19 clustered cells/section; mean ± SD; *p* = 0.023). Single neuroblasts showed a significant increase in the stroke side compared to the contralateral (169 ± 40 vs. 73 ± 25 single cells/section; *p* = 0.0097) (Figure 3E), but the number of neuroblasts in clusters was not significantly different (104 ± 107 vs. 36 ± 28 clustered cells/section; mean ± SD; *p* = 0.211). A possible explanation could be that neuroblasts, unlike transient amplifying progenitor cells, assumed a migratory phenotype during maturation and, in the presence of a stroke, detached from the cluster where they were generated.

## 4. Discussion

Ischemic stroke induces a limited neurogenic response in the striatum. Many of the generated neurons derive from local astrocytes, which initiate neurogenesis in response to the decreased Notch-signaling caused by the stroke. We report here that further reducing Notch-signaling by *Rbpj* deletion recruits more astrocytes and increases neurogenesis approximately 3.5-fold in the striatum following stroke. After such blockade of Notch-signaling, more astrocytes in the striatum initiate the expression of the proneural transcription factor Ascl1 and give rise to a larger number of transit amplifying progenitor cell clusters and neuroblasts. Continuous Notch-signaling hinders astrocytes from entering the neurogenic trajectory in the healthy striatum. Stroke reduces Notch-signaling in astrocytes, but only a minority of astrocytes still initiates neurogenesis. Our finding that blocking Notch-signaling recruits substantially more astrocytes to neurogenesis following stroke suggests that the reduction in Notch-signaling caused by stroke is not of sufficient magnitude to recruit all striatal astrocytes competent to generate neurons.

The increased recruitment of astrocytes to neurogenesis by the combination of stroke and *Rbpj* deletion compared to *Rbpj* deletion alone also suggests that there are pathways other than Notch-signaling that may influence this process. Interestingly, whereas stroke or blocking Notch-signaling in isolation results in the induction of neurogenesis by astrocytes almost exclusively in the medial striatum, the combination results in substantial neurogenesis also in the lateral striatum. It seems possible that the medial striatum, with its proximity to the neurogenic niche in the subventricular zone, is a more neurogenesis-permissive environment with likely higher concentrations of mitogens. It is well known that brain injury results in increased concentrations of several mitogens and neurotrophic factors, which could contribute to the increased recruitment of astrocytes to the neurogenic lineage in the combination of stroke and blocked Notch-signaling. This is further supported by the finding that *Rbpj* deletion alone is insufficient to induce astrocytes in the somatosensory or occipital cortex to give rise to neurons, whereas the combination of inhibition of Notch signaling and a local injury triggers cortical neurogenesis by parenchymal astrocytes [21].

There is currently no regenerative therapy to promote recovery after stroke, and the vast majority of neurons that die are never replaced. Therefore, the generation of new neurons from endogenous astrocytes represents an interesting therapeutic opportunity, in particular, since it may promote neuronal replacement without the need for intracerebral cell transplantation, which carries inherent risks. Our findings suggest that small molecules that inhibit Notch-signaling, which have been used in clinical trials for other indications, should be evaluated for a potential regenerative effect after stroke.

## Supplementary Materials

The following are available online at https://www.mdpi.com/2073-4409/9/7/1732/s1: Figure S1—Neuroblasts from the striatum mature into NeuN+ neurons following stroke, Figure S2—Ascl1^+^/Tomato^+^ cells in the penumbra of stroke maintain the expression of the astrocytic marker S100β. Figure S3—Ascl1^+^/Tomato^+^ cells in the penumbra of stroke express the proliferation marker Ki67 and proliferate to form clusters.
