# Supplementary material for: Blocking Notch-Signaling Increases Neurogenesis in the Striatum after Stroke
Source: Cells. 2020 Jul 20;9(7):1732. doi: 10.3390/cells9071732 (PMC7409130; doi:10.3390/cells9071732)
Supplement: Supplementary file 1 [file cells-09-01732-s001.pdf]

cells MDPI

1. Supplementary figures

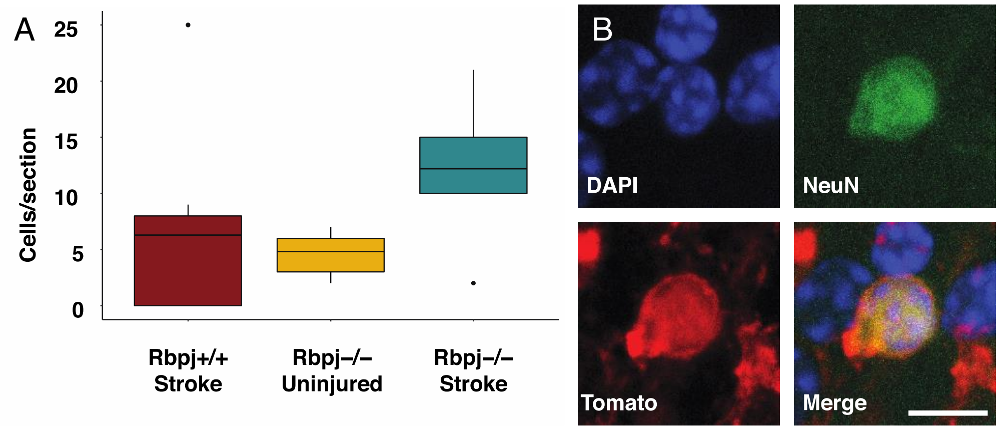

Figure S1. Neuroblasts from the striatum mature into NeuN+ neurons following stroke. (**A**) NeuN+ cells were quantified and compared between the injured hemisphere of *Rbpj*$^{-/-}$ mice (12±7 cells/section; mean±SD), the uninjured hemisphere of the same animals (5±2 cells/section; mean±SD; p=0.125), and the injured hemisphere of *Rbpj*$^{+/+}$ mice (6±9 cells/section; mean±SD; p=0.145). (**B**) Close-up of a NeuN^{+}/Tomato^{+} cell found in the penumbra of the stroke area (scalebar = 10μm).

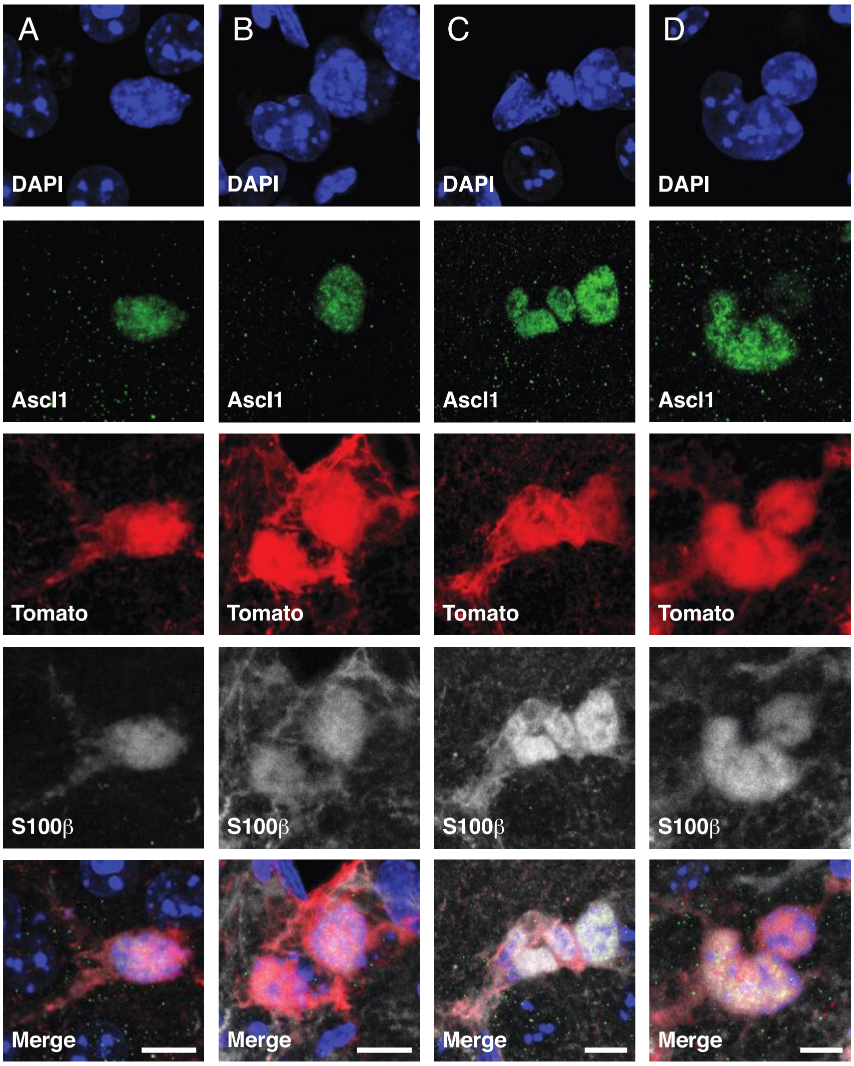

Figure S2. Ascl1⁺/Tomato⁺ cells in the penumbra of stroke maintain the expression of the astrocytic marker S100β: (**A**–**B**) Astrocytes during the first stages of differentiation maintain their typical morphology and expression of the marker S100β while starting to express the proneural transcription factor Ascl1; (**C**–**D**) Ascl1⁺ clusters, which maintain the expression of the astrocyte marker S100β, lose the astrocytic processes and assume a round morphology more reminiscent of transient amplifying progenitor cells (all scalebars = 10μm).

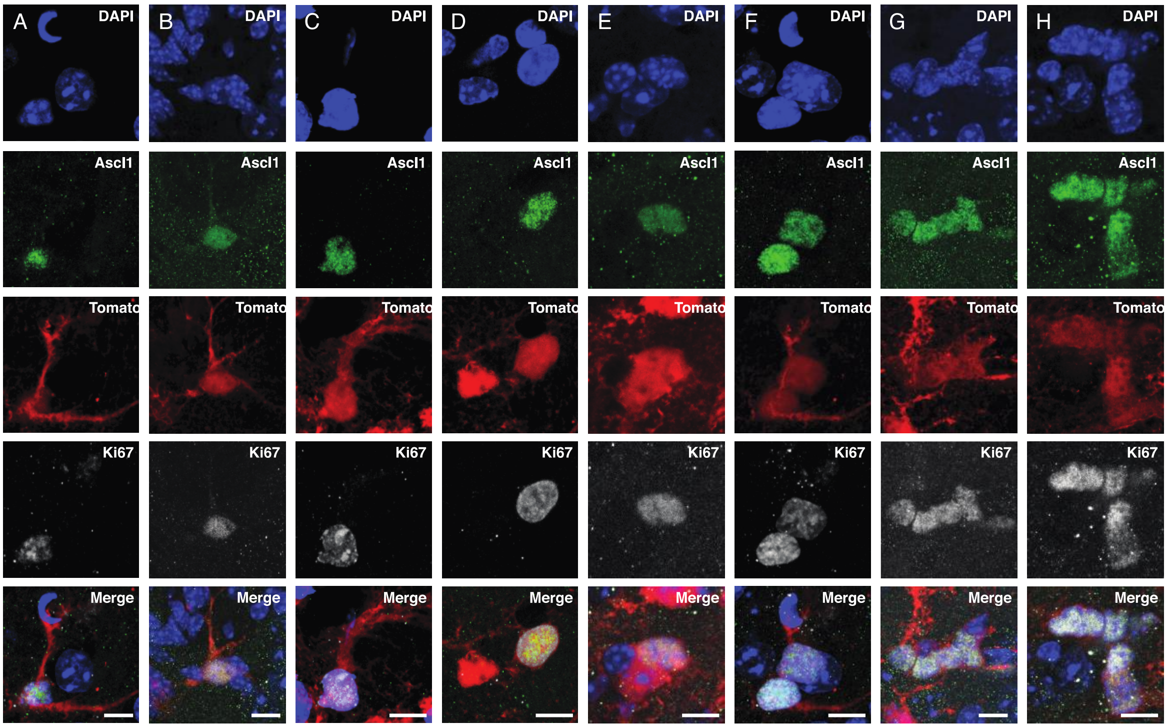

Figure S3. Ascl1⁺/Tomato⁺ cells in the penumbra of stroke express the proliferation marker Ki67 and proliferate to form clusters: (**A–E**) Single Ascl1⁺/Tomato⁺ cells express the cell proliferation marker Ki67 during the first stages of the cluster formation, while they maintain the typical morphology of astrocytes; (**F–H**) Clusters of Ascl1⁺/Tomato⁺ cells lose the astrocyte processes and assume a round shape after the first divisions while continuing proliferation (all scalebars = 10μm).
